# The impact of high polymerization inulin on body weight reduction in high-fat diet-induced obese mice: correlation with cecal *Akkermansia*

**DOI:** 10.3389/fmicb.2024.1428308

**Published:** 2024-08-29

**Authors:** Liping Gan, Yifeng Zhao, Zongbao Zhang, Chenkai Zhao, Jiake Li, Qingyu Jia, Yusu Shi, Peng Wang, Linna Guo, Hanzhen Qiao, Yaoming Cui, Jinrong Wang

**Affiliations:** School of Bioengineering, Henan University of Technology, Zhengzhou, China

**Keywords:** inulin, polymerization degree, obesity, gut microbiota, high fat diet, *Akkermansia*

## Abstract

Obesity presents a significant public health challenge, demanding effective dietary interventions. This study employed a high-fat diet-induced obesity mouse model to explore the impacts of inulin with different polymerization degrees on obesity management. Our analysis reveals that high-degree polymerization inulin (HDI) exhibited a significantly higher oil binding capacity and smaller particle size compared to low-degree polymerization inulin (LDI) (*p* < 0.05). HDI was more effective than LDI in mitigating body weight gain in high-diet induced obese mice, although neither LDI nor HDI affected blood sugar levels when compared to the high-fat diet control group (*p* < 0.05). Both HDI and LDI administrations reduced liver weight and enhanced brown adipose tissue thermogenesis compared to the high-fat diet induced control group (*p* < 0.05). Additionally, HDI suppressed hepatic lipogenesis, resulting in a further reduction in liver triglycerides compared to the high-fat diet-induced obese mice (*p* < 0.05). Notably, HDI improved gut health by enhancing intestinal morphology and modulating gut microbiota structure. HDI administration notably increased the relative abundance of cecal *Akkermansia*, a gut microbe associated with improved metabolic health, while LDI showed limited efficacy (*p* < 0.05 and *p* > 0.05, respectively). These findings underscore the importance of the structural properties of inulin in its potential to combat obesity and highlight the strategic use of inulin with varying polymerization degrees as a promising dietary approach for obesity management, particularly in its influence on gut microbiota composition and hepatic lipid metabolism regulation.

## Introduction

Obesity has emerged as a global health crisis over the past few decades, with rates tripling worldwide in the last half-century (Prillaman, 2023). This surge in obesity has brought with it a myriad of health complications, including fatty liver disease, type 2 diabetes (T2D), etc., contributing to decreased life expectancy and imposing substantial economic burdens on both individuals and societies (Eliby, 2022). While lifestyle modifications aimed at reducing calorie intake and increasing energy expenditure are commonly advocated for obesity prevention and treatment, their long-term efficacy remains limited (Bluher, 2019). Although several FDA-approved drugs for obesity treatment exist, their effectiveness varies among individuals, and there is a paucity of research on their safety and efficacy in adolescents and children (Prillaman, 2023). Hence, the pursuit of safe and effective dietary interventions, particularly those involving dietary fiber, has gained considerable attention.

Inulin, a widely studied dietary fiber, has gained prominence in recent research as an effective dietary intervention to combat obesity and related disorders in both adults and children (Al-Abdullatif and Azzam, 2023; Singh et al., 2018; Visuthranukul et al., 2022). Its appeal lies, in part, in its extraction from readily available sources like chicory and Jerusalem artichokes, which are rich in inulin with relatively simple fructose-based compositions. However, it is important to note that commercial inulin products, particularly those derived from chicory, encompass various types classified based on their polymerization degree (DP). This raises a crucial question: do all types of inulin yield identical anti-obesity effects?

Previous studies have explored the anti-obesity potential of inulin with varying DP, yielding mixed results. Some studies have reported that combining high-fat diets with high DP inulin led to reductions in body and liver weight in mice, whereas low DP inulin yielded no such effects (Du et al., 2020). Conversely, other researchers, utilizing different degrees of polymerized inulin in conjunction with high fat diet (HFD), found no impact on body and liver weight compared to HFD-fed mice (Li et al., 2020). These disparities in findings may be attributed to differences in experimental conditions and the metabolic status of the subjects, particularly the latter.

Crucially, inulin as a fermentable dietary fiber, exerts its anti-obesity properties by profoundly influencing the composition of the intestinal microbiota. The gut microbiome plays a pivotal role in host energy metabolism and is a promising target for weight management due to its intricate connection with obesity. Dysbiosis of the intestinal microbiota is a well-documented consequence of obesity, and it can significantly impact the host’s physical condition (Delannoy-Bruno et al., 2021). Furthermore, the DP of inulin can have varying effects on microbiota. Although several studies have evaluated the impact of inulin with different DP on gut microbiota, many of these studies initiated interventions with high-fat diets and inulin from the start of the study, using mice of normal weight (Du et al., 2020; Hu et al., 2021; Li et al., 2020; Zhu et al., 2019). Given the fact that there are significant differences in gut microbiota and metabolic status between obese and lean individuals, administering inulin combined with a high-fat diet to mice with normal weight may not accurately reflect the effects of inulin on obese individuals. Therefore, further investigation is required to understand the effects and mechanisms of inulin on obese individuals. Additionally, while some studies assessed changes in colonic microbiota following inulin intervention, others utilized fecal samples (Li et al., 2020; Zhu et al., 2017). It is important to explore the effects of various DP of inulin specifically on cecal microbiota, as the cecum serves as the primary microbial fermentation site in mice.

The structural properties of different polymerized inulin types may offer insights into whether they uniformly affect obesity and its underlying mechanisms. In this study, we induced obesity in mice using a high-fat diet and subsequently administered inulin treatment, comparing the properties of inulin with varying DP both *in vitro* and *in vivo*. The study involved in-depth assessments of gut microbiota, liver health, and body weight in response to different polymerized inulin treatments. By examining these critical aspects, we sought to shed light on the potential applications of inulin as a dietary intervention for individuals dealing with obesity and overweight conditions.

## Materials and methods

### Inulin

Low DP inulin (LDI, Orafti P95, purity = 97.8%, 4 < DP <6) and high DP inulin (HDI, Orafti HP, purity = 100%, DP ≥ 23 units) used in this study were sourced from Beneo (Mannheim, Germany). These specific types of inulin were selected based on their purities and DP to ensure accurate and reliable results. Inulin was incorporated into the mice’s feed as part of the experimental procedure.

## Morphological analysis of inulin in the current experiment

The microstructure and morphology of the inulin used in this experiment were examined using a scanning electron microscope (SEM, SU8200, Hitachi, Japan) operated at an accelerating voltage of 3 kV and magnifications of 100×, 500×, and 1,000×. Approximately 0.1 g of inulin powder was affixed to the SEM stub, followed by sputter coating with gold. Subsequently, images were captured using the SEM.

To assess the particle size of the inulin powder, the diameters of 6–8 particles were measured from three images taken at different perspectives, utilizing magnifications of 500× and 1,000× for LDI and HDI, respectively. This analysis was conducted using Fiji (ImageJ-win64).

### Oil absorption capacity

The absorption capacity of inulin for oils was determined using a modified version of the method described by Jeddou et al. (2016). In brief, a reaction system was prepared in a pre-weighed centrifuge tube, where 0.5 g of HDI or LDI were mixed with 10 mL of corn oil. The oil absorption capacity was calculated using the following formula:

Oil Absorption Capacity (g oil/g polysaccharide) = [(weight of the tube contents after draining – weight of the dried polysaccharide) / weight of the dried polysaccharide].

### The effects of inulin with different degree of polymerization on bacteria *in vitro*

The effects of inulin on *Lactobacillus rhamnosus* (ATCC 9595), *L. plantarum* (CICC 24936), and *L. reuteri* (CICC 6119), which were routinely cultivated in either liquid or solid MRS medium, were detected. For evaluating the inhibitory effects of inulin on Opportunistic pathogen, we utilized *Escherichia coli ETEC* (CICC 10667) and *Staphylococcus aureus* (CICC AB 91093), which were also routinely grown in either liquid or solid LB medium. After inoculating approximately 1 × 10^6^ CFU/mL of each of the five bacterial strains in MRS or LB liquid medium, with and without 8% HDI or LDI, the cultures were incubated at 37°C for 24 h. Three replicates were conducted for each treatment and each bacterial strain. After the 24 h incubation period, the optical density of the liquid medium was measured at 600 nm. Subsequently, each bacterial culture was appropriately diluted for colony counting on agar plates.

### Fourier transform infrared spectroscopy of inulin powder

In this experiment, inulin samples with varying DP were subjected to Fourier Transform Infrared Spectroscopy (FTIR) using the Nicolet iS20 instrument from Thermo Scientific, United States. The absorption spectra were recorded across a frequency range spanning from 400 to 4,000 nm. For each analysis, a portion of the inulin powder was carefully positioned on the attenuated total reflectance (ATR) section of the spectrometer. Over 64 scans were conducted, each at a resolution of 4 cm^−1^, and the resulting spectra were averaged to obtain a representative spectrum.

### Animals and treatment

The animal protocols utilized in this study received approval from and were conducted in accordance with the Animal Care and Use Committee of Biological Engineering, Henan University of Technology (no. HAUTETHI-2022-1099).

A total of 32 healthy male C57BL/6 J mice, aged 5 weeks and with similar body weights, were procured from the laboratory animal center of Zhengzhou University for the purpose of this study. These mice were accommodated in the animal facility at Henan University of Technology. Following a one-week acclimatization period, the mice were randomly divided into four groups, each comprising eight mice, ensuring that there were no significant weight discrepancies between the groups. The mice were housed in pairs within cages and were provided with either a low-fat diet (10% of energy from fat, TP 23302) or a high-fat diet (60% of energy from fat, TP 23302), both obtained from Trophic Animal Feed High-Tech Co., Ltd., China. The treatment groups consisted of the following: high-fat diet control group (HFD), Low-fat diet control group (LFD), High-fat diet with LDI treatment group (HFD + LDI), High-fat diet with HDI treatment group (HFD + HDI). To explore the effects of inulin with different degrees of polymerization (DP) in obese mice, we first induced obesity in the mouse model. For the initial 4 weeks, the LFD group was fed a low-fat diet, while the other three groups received the same basal high-fat diet (ingredients listed in Supplementary Table S1). Subsequently, the HFD + LDI and HFD + HDI groups were administered diets containing 8% LDI or HDI, respectively, added to the basal high-fat diet. The HFD group received a diet with 8% microcrystalline cellulose added, while the LFD group continued on the low-fat diet for 6 weeks. The dosage of inulin used in this experiment is based on previous research evaluating the effects of fiber on high-fat diet- induced mice (Patnode et al., 2019).

All mice were housed in a controlled environment maintained at a temperature of 20–25 °C, with a relative humidity of 60%, under a 12-h light–dark cycle. They were given unrestricted access to food and clean water. High-fat food was stored in the refrigerator to preserve its quality, and water bottles were cleaned weekly, with fresh water replenished every 3 days. Body weights of individual mice and feed intake per cage were recorded on a weekly basis. After 10 weeks of treatment, the mice were euthanized via cervical dislocation for sample collection, which included cecal contents, intestines, liver, white adipose tissue, and brown adipose tissue. White adipose tissue was collected using the method described by (Tan et al., 2018). Briefly, the mouse was pinned on its back on a pad, and the abdominal skin was cut without disturbing the muscle or membranes. The exposed adipose tissue on both sides of the skin was abdominal subcutaneous fat (ASF), which was collected and weighed on an analytical balance. The abdominal muscle was then cut to expose the abdominal organs. The fat around the reproductive organs (epididymal fat, EF) was carefully collected and weighed on both sides. The gut was removed to expose the kidneys, and the adipose tissue surrounding the kidneys (perirenal fat, PF) was collected and weighed after removing the adrenal glands. Brown adipose tissue, located at the junction of the back and neck (Lee et al., 2022), was also collected and weighed.

**Figure 1:**
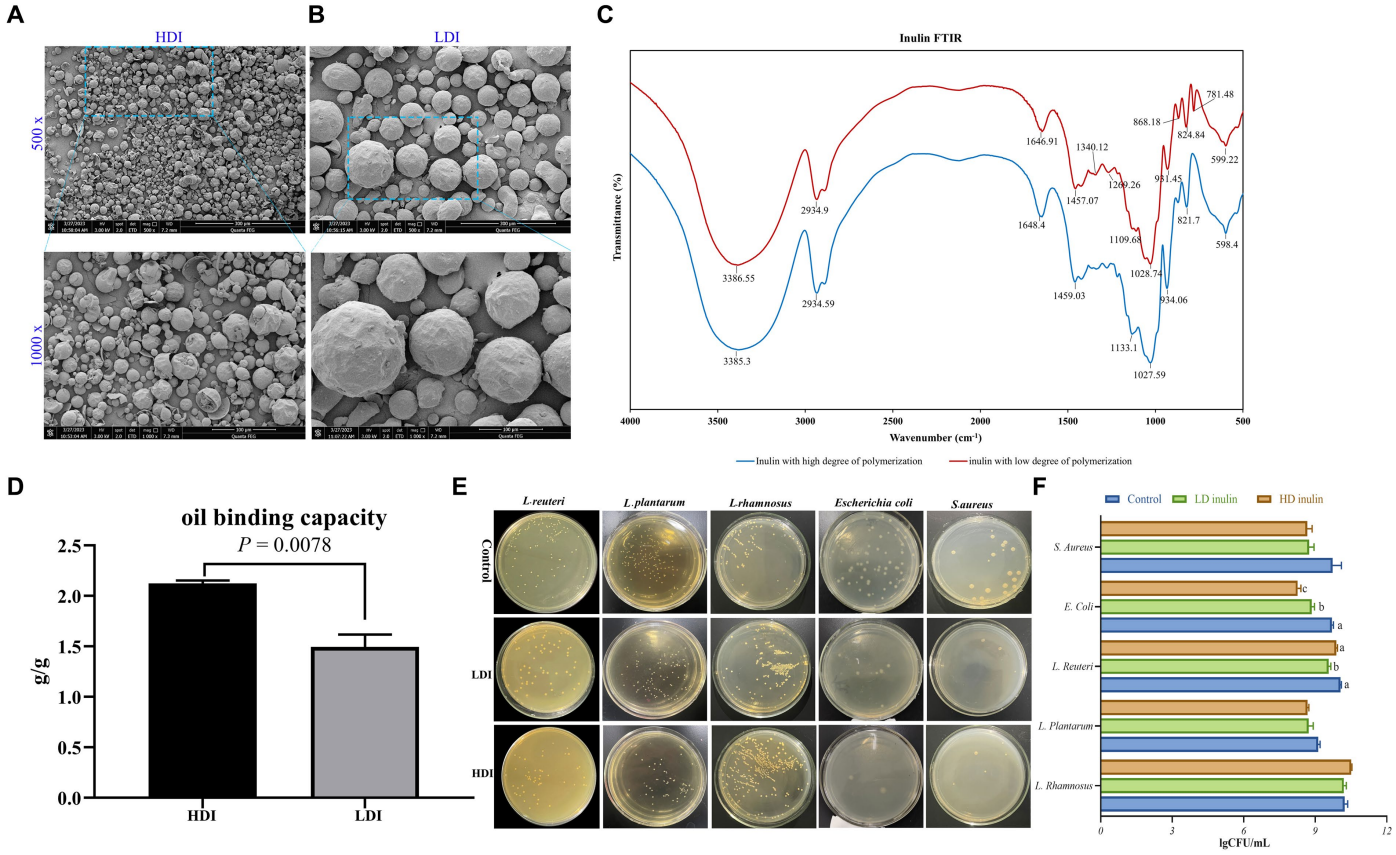
Characterization of low-degree polymerized inulin (LDI) and high-degree polymerized inulin (HDI). **(A)** Scanning electron microscopy (SEM) images of HDI, with 500× magnification in the upper panel and 1,000× magnification in the lower panel; **(B)** SEM images of LDI, with 500 × magnification in upper panel and 1,000 × in the lower panel; **(C)** Fourier transform infrared (FTIR) spectrum of LDI and HDI; **(D)** oil binding capacity of HDI and LDI inulin; **(E,F)** effects of LDI and HDI on the growth of *L. rhamnosus, L. plantarum, L. reuteri, E. coli,* and *S. aureus in vitro*.

### Oral glucose tolerance test of the mice

In order to assess the disposal of an orally administered glucose load over time in mice subjected to various treatment regimens, the OGTT method was employed in this study, as previously described by (Nagy and Einwallner, 2018).

Briefly, at the conclusion of the ninth week, the mice were transferred to fresh bedding and subjected to an overnight fast lasting 12 h, during which they retained access to drinking water. Subsequently, a small drop of blood sample (~3 μL) was obtained by carefully removing 1 mm from the tail tip for the measurement of basal blood glucose levels using a glucometer (Yuehao I 720, Yuwell, Jiangsu, China). Following the basal measurement, a glucose solution (1 g glucose/kg body weight) was administered directly into the stomach of each mouse via a feeding needle. At time intervals of 15, 30, 60, 90, and 120 min after the administration of glucose, blood glucose levels were measured using the glucometer for each individual mouse.

### mRNA expression detected by quantitative real-time (qRT)-PCR

Total RNA was extracted from liver and brown adipose tissue using Freezol (R711, Vazyme, Nanjing, China) according to the manufacturer’s instructions. The quality and quantity of the RNA were assessed using a NanoDrop 2000. The PrimeScript RT reagent kit with gDNA eraser (TaKaRa, Dalian, China) was used for the reverse transcription according to the manufacturer’s instructions. qRT-PCR was performed in duplicate on a PCR System (qTOWER^3^, Analytik Jena, Germany) with the Universal SYBR qPCR Master Mix (Vazyme, Nanjing, China). The primer sequences used to detect the gene expression are listed in Supplementary Table S2. Gene expression was normalized to the expression of the housekeeping gene beta-actin using comparative 2^-ΔΔCT^ method, as described previously (Livak and Schmittgen, 2001).

### Hematoxylin and eosin (H&E) staining and Oil Red O staining

The small intestinal segments and liver, which were fixed in 4% paraformaldehyde, underwent a series of ethanol dehydration steps before being embedded in paraffin. Subsequently, the tissues were sectioned to a thickness of 8 μm and subjected to hematoxylin and eosin staining. Microscopic images of the liver and intestine tissues were acquired using a microscope (RVL-110-G, Echo Laboratories, United States), and villus height and crypt depth of the small intestine were measured based on magnified images.

For Oil Red O staining, the liver samples underwent a series of dehydration steps in different concentration of sucrose solutions, were then embedded in Optimum Cutting Temperature, quick-frozen in liquid nitrogen, and stored at −80°C overnight prior to sectioning in a cryostat (CM1850 UV, Leica Biosystems, United States). Oil Red O staining was performed in accordance with the procedure outlined by Cui et al. (2017). Following staining, photographs of the stained tissues were captured using a microscope (RVL-110-G, Echo Laboratories, United States).

### Triglycerides contents detection in the liver

The triglycerides contents in the liver of the mice were detected using the triglycerides kit (BC0625, Solarbio Life Sciences, Beijing, China) according to the manufacturer’s instructions.

### Pyrosequencing of cecal microbiota

DNA samples were extracted from cecal digesta using QIAamp DNA Stool Mini Kits (Qiagen Inc., Hilden, Germany) following the manufacturer’s instructions. Subsequently, gel electrophoresis was performed to assess the concentration and purity of the DNA samples. Amplification of microbial 16S rRNA gene sequences was achieved using universal primers designed for the V3-V4 region. The resulting PCR products were subjected to analysis through 2% gel electrophoresis and subsequently purified using the QIAquick Gel Extraction Kit (Qiagen Inc., Hilden, Germany).

Pyrosequencing for 16S rRNA was conducted utilizing the Illumina Novaseq platform (Illumina, San Diego, United States). All procedures were carried out by Biomarker Technologies (Beijing, China). Following quality filtering via Cutadapt (Version 1.9.1) and the removal of chimeric sequences using the UCHIME algorithm (version 8.1), clean reads were obtained. Subsequently, the clean reads were clustered into operational taxonomic units (OTUs) using USEARCH (version 10.0) with a similarity threshold exceeding 97%. These OTUs were then subjected to taxonomy-based analysis using the RDP algorithm in conjunction with the Greengenes database (version 13.5).

### Statistical analysis

Statistical significance was assessed utilizing Student’s *t*-test, one-way ANOVA, or two-way ANOVA for comparisons involving multiple groups. For pairwise comparisons between two groups, the Tukey *post-hoc* test was employed. Data analysis was conducted using Excel or Prism 8. A significance threshold of *p* < 0.05 was applied to determine statistical significance.

## Results

### Morphology of inulin with different molecular weights under SEM

The morphology of inulin with various DP is illustrated in Figures 1A,B. Inulin sourced from chicory presents itself as spherical particles, albeit with some variation in particle size even within a single type of inulin. There were notable distinctions when comparing the two types of inulin, the variant with LDI (Figure 1B) exhibits larger particle sizes than the inulin with HDI (Figure 1A).

**Figure 2:**
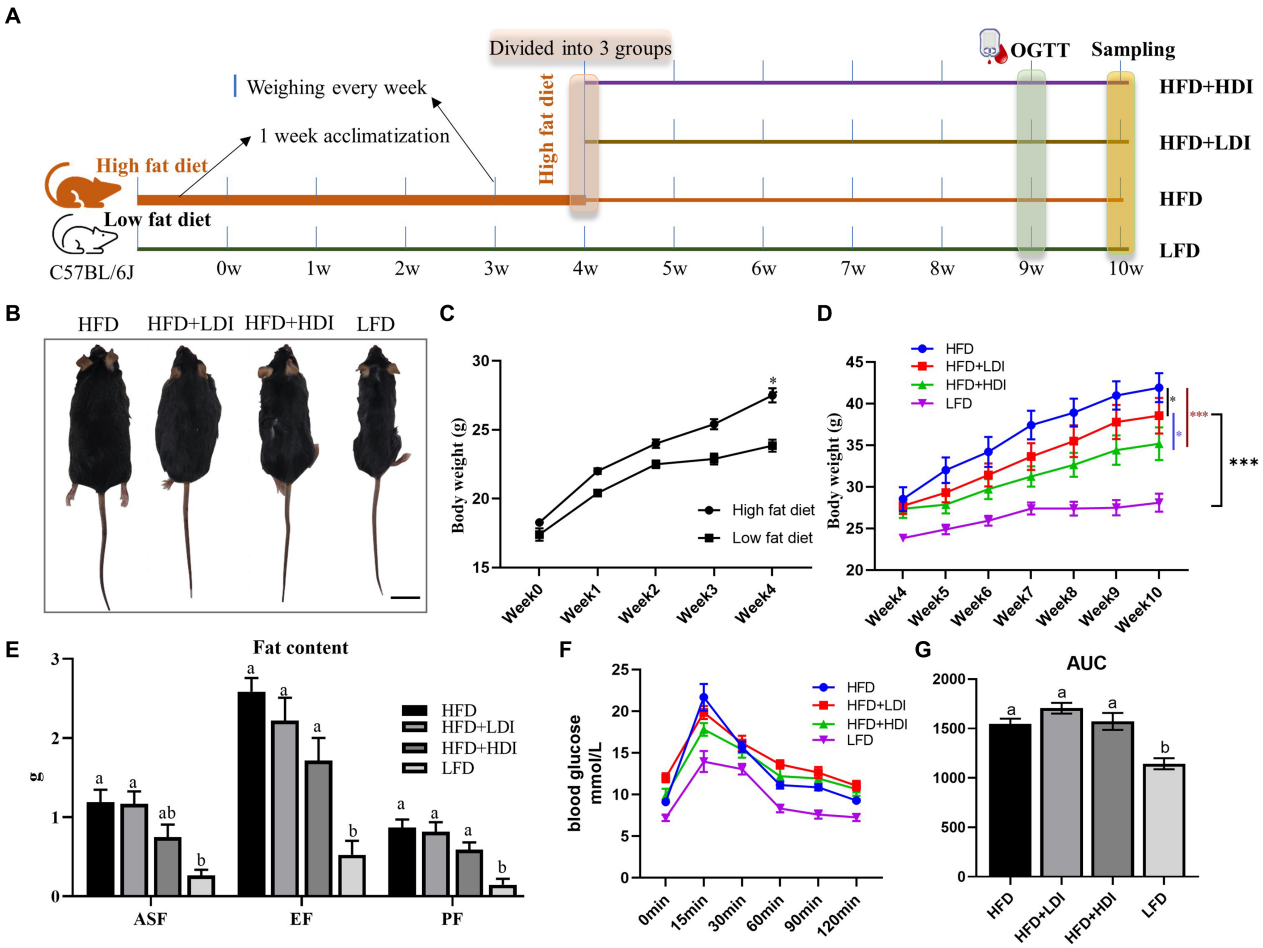
Effects of different polymerization degrees of inulin on body weight and glucose tolerance in obese mice. **(A)** Experimental design of the animal study. **(B)** Representative images of mice in each treatment group at the end of the study. **(C)** Changes in body weight of mice after 4 weeks of high-fat diet treatment (*n* = 24 in the high-fat diet group, *n* = 8 in the low-fat diet group). **(D)** Changes in body weight of mice during the 5th to 10th weeks (*n* = 8). **(E)** Fat contents of mice in different treatment groups (*n* = 8). ASF, abdominal subcutaneous fat; EF, epididymal fat; PF, perirenal fat; **(F)** Glucose levels during the oral glucose tolerance test (OGTT). After an overnight fast, glucose (mg/dL) levels were measured in the fasting state and at 15, 30, 60, 90, and 120 min after administering glucose solution orally via gavage (1 g glucose/kg) (*n* = 8). **(G)** Glucose area under the curve (AUC) during OGTT of mice in different treatment groups. Results are mean ± SEM. *p < 0.05, **p < 0.01, ***p < 0.001. Within each panel, different letters indicate significant changes at *p* < 0.05. Statistical analysis in **(D)** was performed using two-way ANOVA and Tukey’s *post-hoc* test, while in other cases, one-way ANOVA and Tukey’s *post-hoc* test were applied.

Upon magnification of the inulin particles by 1,000 times, it becomes evident that the average particle size of LDI is approximately five times greater than that of HDI (Figures 1A,B, Supplementary Table S3). It is worth noting that while LDI is readily soluble in water, HDI is virtually insoluble in cold to warm water but can be dissolved in hot water.

### Fourier transform infrared spectroscopy of inulin powder

The specificity of carbohydrates arises from the geometry of the O-H groups and the configuration of C-O, C-C, and C-H bonds within the carbon backbone (Coates, 2000). As depicted in Figure 1C, HDI inulin exhibited similar infrared spectra to LDI. Both types of inulin display characteristic band spectra around 3,385, 2,934, and 1,648 cm^−1^, corresponding to O-H stretching, C-H stretching, and O-H deformation, respectively. In accordance with the FTIR analysis conducted by Akram and Garud (2020) and Chikkerur et al. (2020), vibrations within the spectral range of 1,500–900 cm^−1^ primarily result from C-H, O-H, C-O-C bending, C-O-C, and C-O stretching modes within oligo- and polysaccharides.

Although the peak intensity differs between the two types of inulin (see Supplementary Table S4), their peak positions in the spectral range of 1,500 to 900 cm^−1^ remain similar (Figure 1C). Of particular note is the presence of more peaks in the spectrum of LDI compared to HDI within the spectral range of 600–900 cm^−1^, particularly at 781.48 cm^−1^, representing CH_2_ bending (Coates, 2000). The similarity in peak positions at 3386, 2,934, and 1,647 cm^−1^ between LDI and HDI inulin suggests that similar functional groups are present in both.

### Oil binding capacity two types of inulin *in vitro*

The oil holding capacity is a critical characteristic and a technological property closely associated with the chemical structure of polysaccharides. As depicted in Figure 1D, HDI exhibits a significantly greater oil binding capacity (*p* = 0.0078, 2.123 vs. 1.494 g/g) compared to LDI.

### Body weight and plasma glucose levels of the mice during the experiment

The experimental outline for each group is illustrated in Figure 2A. Following 4 weeks of high-fat diet treatment, mice in the high-fat diet group exhibited significantly higher body weights compared to those on the low-fat diet (*p* < 0.05, Figure 2C). By the fourth week, the body weights of mice in the various high-fat diet groups were statistically similar (*p* > 0.05). Subsequently, after administering inulin with different DP to the high-fat diet mice for 6 weeks, significant reductions in body weight were observed in both the LDI (*p* < 0.05) and HDI (*p* < 0.001) inulin groups. Furthermore, the body weight of mice in the HFD + HDI group was significantly lower than that in the HFD + LDI group (*p* < 0.05). Despite the reductions in body weight induced by the addition of inulin to the HFD groups, the body weight in these groups remained higher than that of the LFD group (*p* < 0.001, see Figures 2B,D).

High-fat diet consumption significantly increased the weight of ASF, EF, and PF in mice (*p* < 0.001, Figure 2E) when compared to mice in LFD. Mice in the HFD + HDI group experienced reductions in ASF, EF, and PF contents by approximately 37, 34, and 32%, respectively, when compared to mice in the HFD group. However, there were minimal differences between mice in the HFD and HFD + LDI groups concerning fat contents.

Plasma glucose levels of the mice are depicted in Figure 2F. High-fat diet consumption elevated fasting blood glucose levels in comparison to the LFD group. Moreover, blood glucose levels had not returned to baseline even 120 min after oral administration of glucose. Supplementation with inulin of different polymerization degrees did not ameliorate this phenomenon. Notably, mice in the HFD, HFD + LDI, and HFD + HDI groups exhibited higher area under the curve (AUC) values than mice in the LFD group, suggesting that the addition of inulin had minimal effects on blood glucose levels (Figure 2G).

### Inulin reduces lipid content in the liver of HFD-induced mice

**Figure 3:**
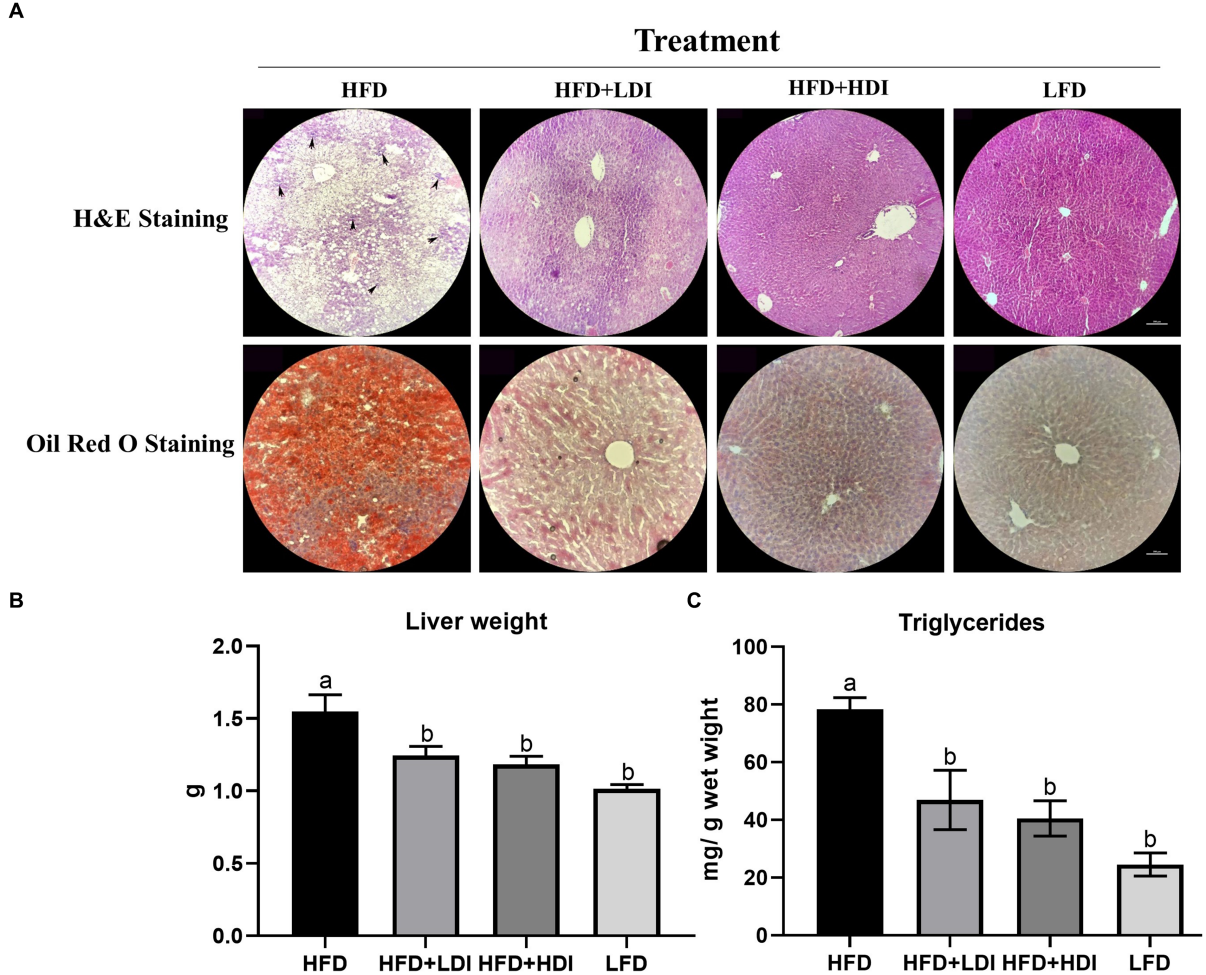
Effects of different polymerization degrees of inulin on liver health of high-fat diet and low-fat diet mice. **(A)** Hematoxylin and eosin (H&E) staining and Oil Red O staining of the liver of mice in different treatment groups. The arrowheads indicate inflammatory cell infiltration. **(B)** Liver weight of mice in different treatment groups. **(C)** Triglyceride levels in different treatment groups (*n* = 8). Within each panel, groups without a common letter indicate significant differences at *p* < 0.05. Scale bar, 500 μm.

As demonstrated in Figure 3B, mice in the HFD group exhibited a significant increase in liver weight compared to mice in the LFD group (*p* = 0.001). Notably, both HFD + HDI and HFD + LDI resulted in reduced liver weight when compared with the HFD group (*p* < 0.05). This reduction in liver weight may be attributed to the decreased triglyceride content in the liver, as illustrated in Figures 3A,C.

High-fat diet consumption led to fat accumulation in the liver, as evident in H&E and Oil Red O staining in Figure 3A. Furthermore, compared to the HFD + HDI, HFD + LDI, and LFD groups, the livers of mice in the HFD group exhibited greater immune cell infiltration, as indicated by the arrowheads in Figure 3A, indicative of increased liver inflammation. However, supplementation with high- and low- DP inulin effectively reduced the size of lipid droplets and inflammation in the liver.

**Figure 4:**
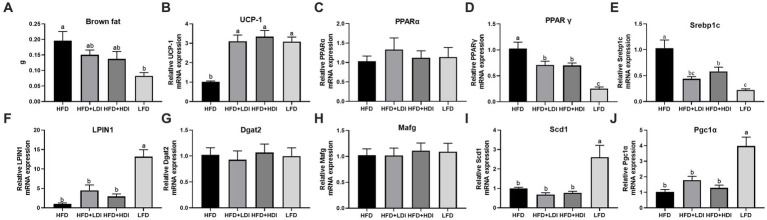
Effects of different polymerization degrees of inulin on gene expression in brown tissue and liver of high-fat diet mice. **(A,B)** Weight of brown fat tissue in different treatment groups. **(B)** Uncoupling protein 1 (UCP-1) gene expression in brown fat tissues. **(C-J)** Hepatic gene expression of PPARα, PPARγ, Srebp1, LPIN1, Dgat2, Mafg, Scd1, and Pgc1α (*n* = 8). Within each panel, groups without a common letter indicate significant differences at *p* < 0.05. HFD, high fat diet; HFD + HDI, high fat diet with high polymerized degree inulin; HFD + LDI, high fat diet with low polymerized degree inulin; LFD, low fat diet.

To elucidate the mechanisms underlying the reduction of fat accumulation in both liver and adipose tissues by inulin, we assessed the genes related to fat metabolism in both brown adipose tissue and the liver of mice subjected to different treatment regimens. As presented in Figures 4A,B, the weight of brown adipose tissue in the LFD group significantly decreased compared to that in the HFD mice (*p* < 0.05). Furthermore, mice fed both LDI and HDI inulin exhibited similar brown adipose tissue weights to those in the HFD groups (*p* > 0.05). Concurrently, the mRNA expression of uncoupling protein 1 (*UCP-1*) in brown adipose tissue was significantly higher in the HFD + LDI, HFD + HDI, and LFD groups than in the HFD group (*p* < 0.05).

Eight mRNA expressions related to hepatic lipid metabolism were examined in this study (Figures 4C–J), with only two genes—peroxisome proliferator-activated receptor γ (*PPARγ*, Figure 4D) and sterol regulatory element-binding protein 1 (*SREBP1*, Figure 4E)—being reduced by inulin administration. In contrast, five genes were influenced by dietary fat content, including reduced mRNA expression of *PPARγ* and *SREBP1* and increased mRNA expression of *LPIN*, stearoyl-coenzyme A desaturase 1 (*SCD1*), and PPARγ coactivator 1α (*PGC1α*) by the LFD compared to the HFD group (*p* < 0.05).

### Different polymerization degrees of inulin improve the intestinal health of obese mice

**Figure 5:**
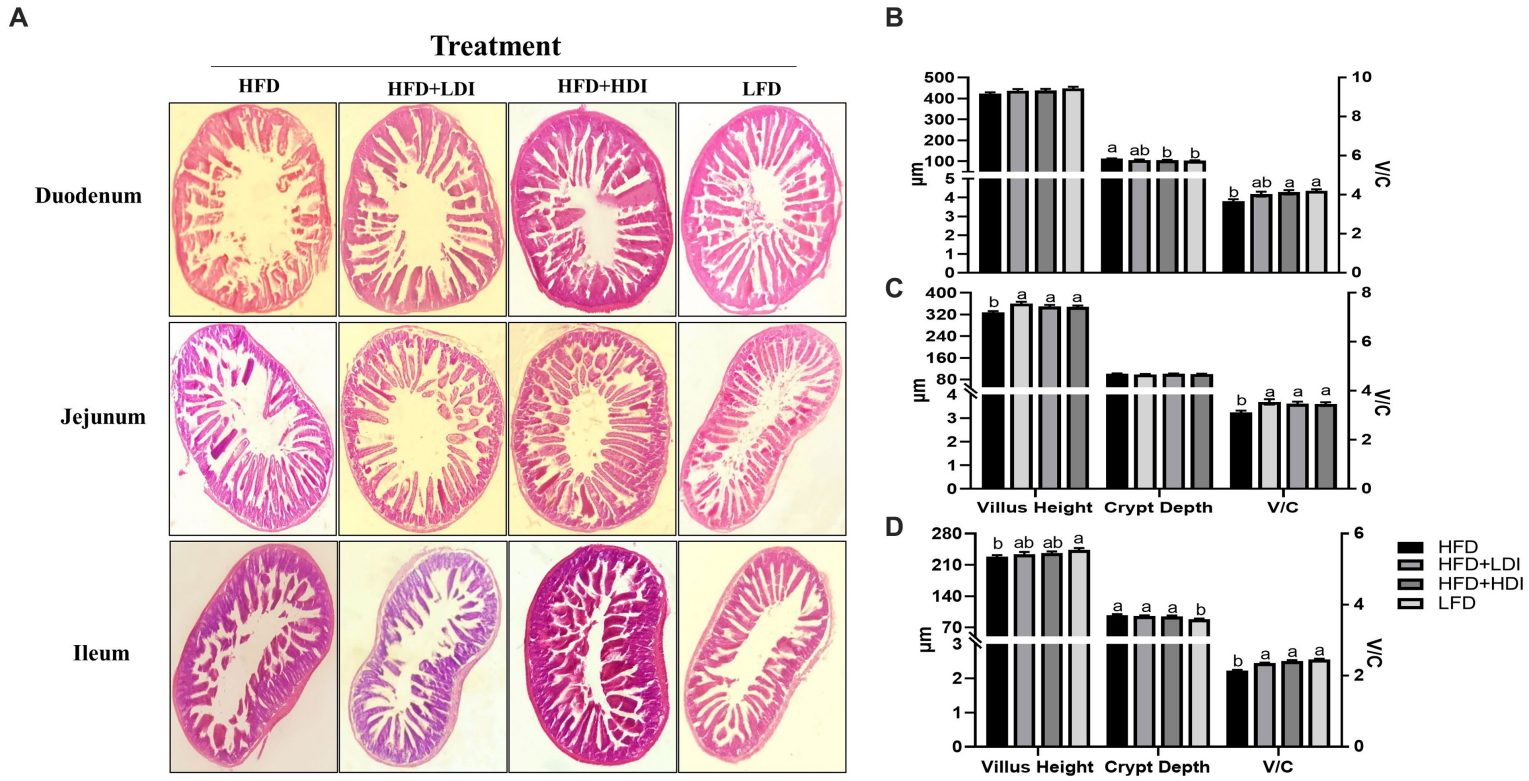
Effects of different polymerization degrees of inulin on intestinal health of high-fat diet-induced obese mice. **(A)** Morphology of duodenum, jejunum, and ileum in mice with different treatments. **(B–D)** Villus height, crypt depth, and the ratio of villus height to crypt depth (V/C) of duodenum, jejunum, and ileum, respectively, in mice with different treatments (*n* = 8). Within each panel, groups without a common letter indicate significant differences at *p* < 0.05. HFD, high fat diet; HFD + HDI, high fat diet with high polymerized degree inulin; HFD + LDI, high fat diet with low polymerized degree inulin; LFD, low fat diet.

Figure 5A displays the results of H&E staining of the small intestine in mice subjected to various treatment regimens. High-fat diet consumption induced atrophy of the small intestinal villi, resulting in reduced villus height in both the ileum and jejunum of the mice (*p* < 0.05) in comparison to mice in LFD group. Notably, the administration of HDI significantly increased the V/C ratio in the duodenum, jejunum, and ileum (*p* < 0.05, Figures 5B–D) of the mice when compared to those in the HFD groups. Furthermore, LDI also elevated the V/C ratio in the duodenum and ileum in comparison to mice in the HFD groups (*p* < 0.05). The supplementation of inulin effectively enhanced the morphology of the intestine in high-fat diet-induced obese mice.

### The effects of inulin on the microbiota both *in vitro* and *in vivo*

As depicted in Figures 1E,F, the supplementation of inulin led to a significant reduction in the abundance of *E. coli* when compared to the basic LB control medium (*p* < 0.001). Notably, HDI inulin exhibited a more potent inhibitory effect on *E. coli* than LDI inulin (*p* = 0.03).

Furthermore, the inclusion of inulin in the LB medium demonstrated a tendency to reduce the abundance of *S. aureus* (*p* = 0.059). The supplementation of 8% inulin had minimal impact on the growth of *L. plantarum* and *L. rhamnosus*. However, it was observed that the addition of 8% LDI inulin significantly reduced the abundance of *L. reuteri* (*p* = 0.019), whereas HDI had no discernible effect on *L. reuteri*.

To investigate the impact of inulin on cecal microbiota in obese mice, we employed sequencing of the bacterial 16S rRNA V3 + V4 region. Following data processing, a total of 331 operational taxonomic units (OTUs) were clustered and generated, guided by the principle of over 97% similarity, based on 1,139,609 sequencing reads. The sequencing depth was sufficient to encompass (>99%) all OTUs present in the cecum digesta, providing a nearly comprehensive representation of the overall microbial species richness, as evidenced by rarefaction and ranked abundance (Supplementary Figure S1).

**Figure 6:**
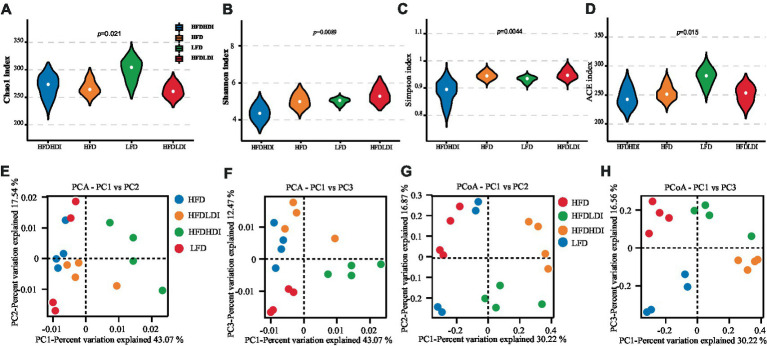
Effects of different polymerization degrees of inulin on the diversity of cecal microbiota in mice with different treatment groups. **(A–D)** Chao1 index, Shannon index, Simpson index, and ACE index of cecal microbiota. **(E,F)** Principal component analysis (PCA) at PC1 and PC2, and PC1 and PC3 of cecal microbiota of the mice. **(G,H)** Principal coordinates analysis (PCoA) at PC1 and PC2, and PC1 and PC3 of cecal microbiota (*n* = 4). HFD, high fat diet; HFD + HDI, high fat diet with high polymerized degree inulin; HFD + LDI, high fat diet with low polymerized degree inulin; LFD, low fat diet.

Differential analysis of α-diversity metrics among groups revealed a significant impact of dietary fat on cecal microbial richness. This was indicated by lower Chao1 and ACE indices in mice from the HFD, HFD + LDI, and HFD + HDI groups compared to those in the LFD treatment group (*p* = 0.021 and 0.015, respectively; Figures 6A,B). Importantly, the supplementation of inulin did not restore microbial richness in the ceca of the mice. Intriguingly, mice administered HDI inulin exhibited lower cecal microbiota diversity, as evidenced by reduced Shannon and Simpson indices compared to those in the HFD and HFD + LDI groups (*p* = 0.0089 and 0.0044, respectively; Figures 6C,D).

For a deeper exploration of beta diversity within cecal microbiota across different treatment groups, we conducted principal components analysis (PCA) and principal coordinates analysis (PCoA) of OTUs in each sample. As illustrated in Figures 6E,F, the bacterial communities of mice in the HFD + HDI group were distinct from those in the HFD and LFD groups. Additionally, ANOSIM analysis on OTUs and other taxonomic levels revealed that the differences between inter-group compositions were more significant than those within the same group, with *p* < 0.05 (Supplementary Figure S2).

**Figure 7:**
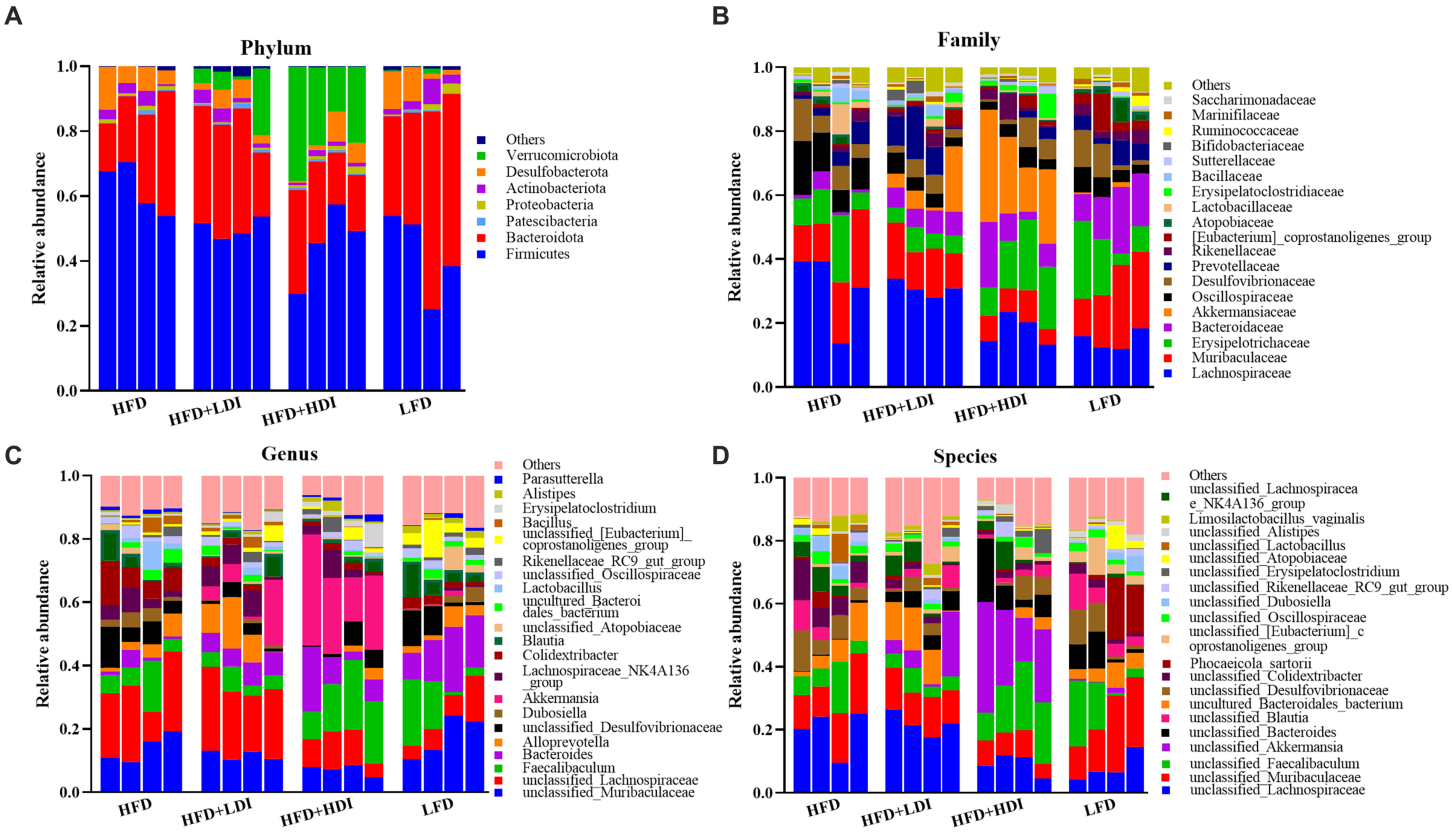
Relative abundance of cecal microbiota at phylum **(A)**, family **(B)**, genus **(C)**, and species **(D)** levels in mice under different treatments (*n* = 4).

Figure 7 presents the relative abundance of cecal microbiota in mice, focusing on phylum and family levels (more than 0.5%) and genus and species levels (more than 1%). The dominant phyla in the cecum of mice were Bacteroidetes and Firmicutes, accounting for 87.7, 82.5, 68.1, and 86.9% in the HFD, HFD + LDI, HFD + HDI, and LFD groups, respectively (Figure 7A). Interestingly, the abundance of Verrucomicrobiota in the cecum of mice in the HFD + HDI group was notably higher than that in other groups (*p* = 0.009, Figure 7A and Supplementary Table S5).

At the family level, the most dominant bacterial families in the cecal contents of the mice from different treatment groups were Lachnospiraceae, Muribaculaceae, Erysipelotrichaceae, Bacteroidaceae, and Akkermansiaceae, collectively accounting for approximately 66% of the bacterial abundance at the family level (Figure 7B). The abundance of Akkermansiaceae, a member of the Verrucomicrobiota phylum, was significantly higher in mice from the HFD group compared to other treatment groups (*p* = 0.021, Supplementary Table S5).

The abundance of cecal bacteria at the genus and species levels also reflected differences in microbiota composition among all treatments. The increased abundance of the *Akkermansia* genus (*p* = 0.013, Figure 7C and Supplementary Table S5) and *unclassified_Akkermansia* species (*p* = 0.017, Figure 7D and Supplementary Table S5) in the HFD + HDI group aligns with the increased presence of the Verrucomicrobiota phylum and the Akkermansiaceae family.

### Identification of microbiota composition in different treatments and correlation analysis

**Figure 8:**
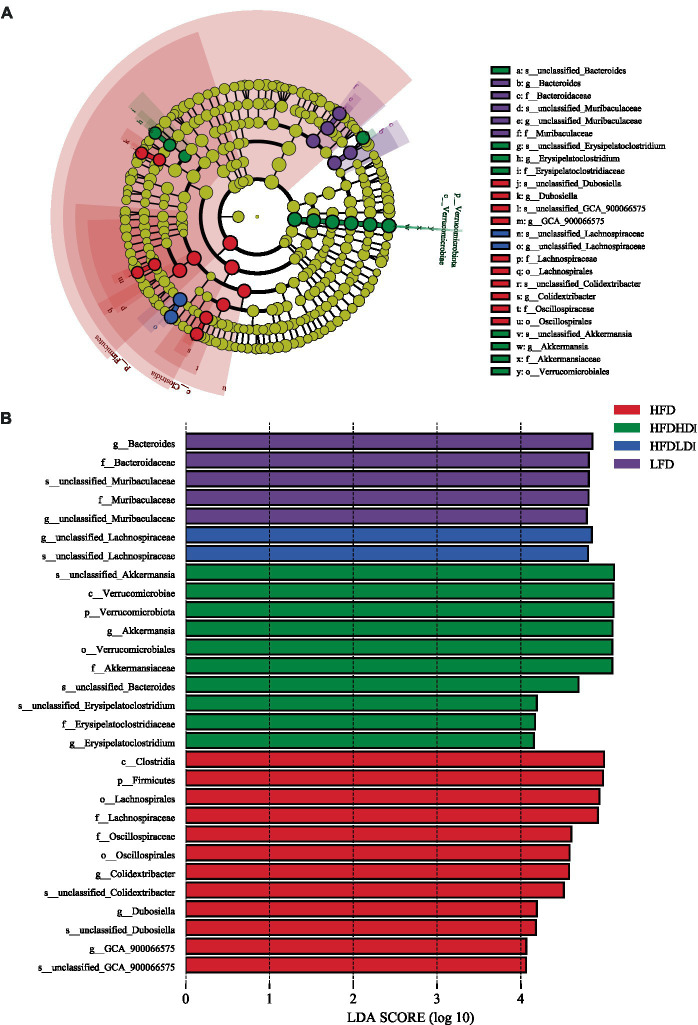
Dominant bacteria in the cecum of mice under different treatments. **(A)** Cladograms representing the LEfSe results for different treatment groups. **(B)** LDA scores of gut microbiota in different groups. HFD, high fat diet; HFD + HDI, high fat diet with high polymerized degree inulin; HFD + LDI, high fat diet with low polymerized degree inulin; LFD, low fat diet.

To further unveil the microbiota with statistically significant differences between different groups, we employed Linear Discriminant Analysis Effect Size (LEfSe) analysis. The cladogram in Figure 8A illustrates the biomarkers in different treatment groups of mice at various taxonomic levels. Additionally, Figure 8B provides a detailed representation of microbiota with LDA scores exceeding 4. At the phylum level, Verrucomicrobiales emerged as the highly abundant microbiota in the HFD + HDI group, while Firmicutes prevailed as the highly abundant microbiota in the HFD group. On the gene level, *Bacteroides*, *unclassified_Lachnospiraceae*, *Akkermansia*, and *Colidextribacter* were identified as highly enriched in the cecal microbiota of mice in the LFD, HFD + LDI, HFD + HDI, and HFD groups, respectively.

**Figure 9:**
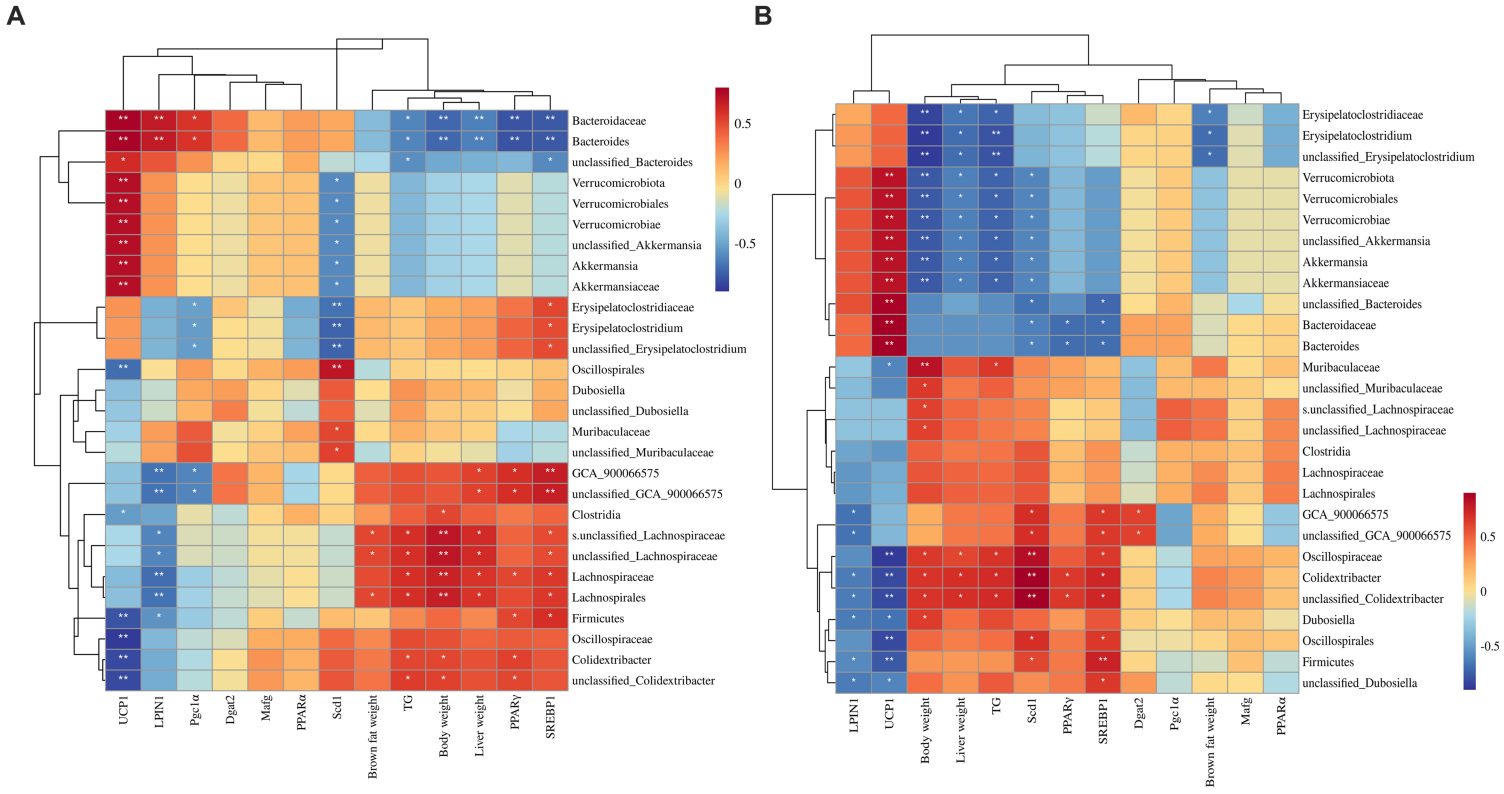
Correlation between biomarker microbiota and other fat-synthesis related parameters. **(A)** Spearman’s correlation conducted using all treatment group data, including the low-fat diet group. **(B)** Spearman’s correlation conducted using data from the three high-fat diet groups. ^*^p < 0.05, ^**^p < 0.01.

To delve deeper into the relationships between these microbiota in different treatments and their potential functions in mice, Spearman correlation analysis was conducted between microbiotas that are distinguishably distributed across different treatment groups and various indexes related to mouse body weight. As depicted in Figure 9A, in the correlation analysis across the four treatment groups, the microbiota in the LFD group, notably Bacteroides, exhibited a strong positive association with gene expression of *UCP1* and *LPIN1*. Conversely, it displayed a highly negative association with body weight, liver weight, liver TG contents, and gene expression of *PPARγ* (*p* < 0.01). In contrast, microbiota highly abundant in the HFD group, such as Lachnospirales, Lachnospiraceae, and *Colidextribacter*, displayed a strong positive correlation with body weight (*p* < 0.05). However, Firmicutes, Oscillospiraceae, and *Colidextribacter* exhibited a highly negative correlation with gene expression of *UCP1* (*p* < 0.05). The microbiota that was notably abundant in the HFD + HDI group, such as Verrucomicrobiota and *Akkermansia*, exhibited a strong correlation with *UCP1* and a negative correlation with *Scd1* gene expression (*p* < 0.05).

**Figure 10:**
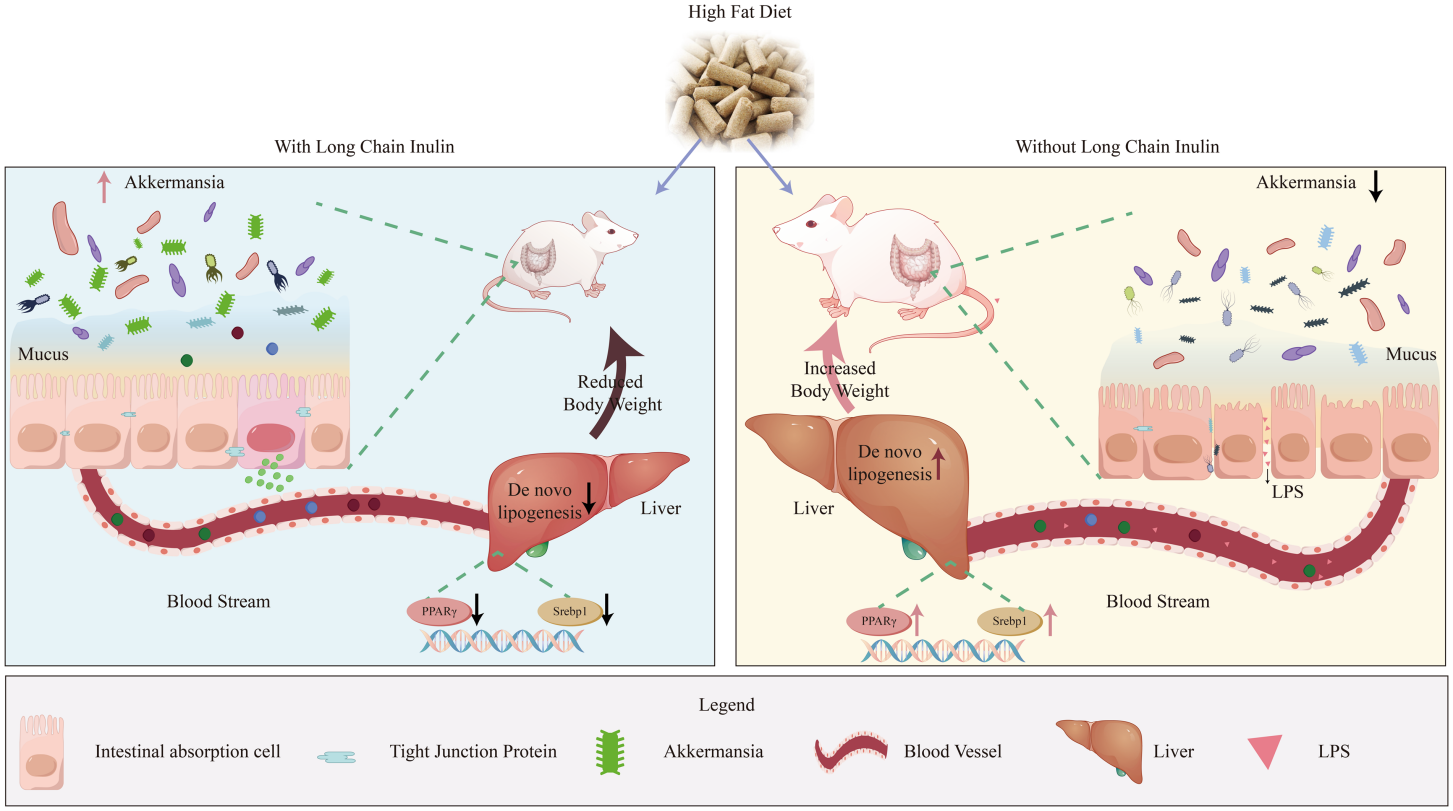
Long-chain inulin reduces body weight in high-fat dietinduced obese mice by inhibiting liver lipogenesis and modulating cecal microbiota composition, particularly increasing the abundance of Akkermansia.

Furthermore, Spearman correlation analysis was conducted among the high-fat diet treatment groups (HFD, HFD + LDI, and HFD + HDI) to examine possible associations of microbiota in the inulin treatment group with other indexes. As shown in Figure 9B, biomarker microbiota in the HFD + HDI group, including *Erysipelatoclostridium*, Verrucomicrobiota, and *Akkermansia*, demonstrated strong negative associations with body weight, liver weight, and liver TG contents (*p* < 0.05). These same microbiota were also positively associated with *UCP1* (*p* < 0.05). Conversely, Oscillospiraceae and *Colidextribacter* in the HFD group displayed strong positive associations with body weight, liver weight, *PPARγ*, and hepatic TG contents, while showing a negative association with *UCP1* gene expression (*p* < 0.05) (Figure 10).

## Discussion

The biochemical functionality of inulin is intricately linked to its structural attributes. Consequently, comprehending the characteristics and functional traits of inulin with varying polymerization becomes imperative for their multifaceted applications across diverse conditions. The DP, often referred to as chain length, wields substantial influence over the biological functions of inulin. In our investigation, we observed that HDI exhibited near-insolubility in water at 25°C, while LDI readily dissolved in water. This observation aligns with prior research findings that have consistently demonstrated a decrease in inulin solubility as the DP increases (Mensink et al., 2015). A closer examination of their morphology revealed that LDI powder exhibited a larger apparent particle size than HDI. The solubility of inulin is closely related to its molecular conformation and DP (Barclay et al., 2010). LDI is more flexible and can adopt a range of conformations and orientations. This flexibility is reflected in the additional peak observed in the FTIR spectra at 781.49 cm^−1^, which corresponds to the hydroxyl functional group. The increased flexibility of LDI may result in different vibrational modes of the OH group, leading to the observed extra peak. Moreover, the presence of hydrophilic functional groups and the flexibility of LDI enhance its ability to form hydrogen bonds and other molecular interactions (Mensink et al., 2015), partially explaining its solubility. In contrast, HDI consists of a larger number of fructose units, forming an extensive hydrogen-bonding network that creates a nearly crystalline structure. This can result in a more rigid and extended molecular configuration, limiting its flexibility and interactions with other molecules. Consequently, water molecules have difficulty penetrating and surrounding HDI molecules, leading to their limited solubility. Additionally, inulin is known to exhibit intermolecular forces (French, 1989), which may further stabilize the structure of HDI and reduce its affinity for water. The larger particle size observed in LDI may be attributed to the tendency of shorter chains to agglomerate. Furthermore, due to the presence of hydrophilic groups in LDI, exposure to air may cause water molecules to interact with the hydrophilic groups on the surface of LDI, causing it to swell and appear larger when observed under a microscope.

Many polysaccharides and dietary fibers are characterized by their oil binding capacity, a property that plays a crucial role in delaying the digestion and absorption of dietary fats. This delay in fat absorption can contribute to the prevention of obesity (You et al., 2022). Consequently, inulin’s ability to bind to dietary oils can slow down the digestion and absorption of fats, potentially leading to increased fat excretion. This mechanism may explain the reduction in body weight observed in high-fat diet-induced obese mice following inulin administration. Furthermore, it is worth noting that HDI exhibits a higher oil binding capacity than LDI. This difference in oil binding capacity could account for the superior effects of HDI in reducing body weight compared to LDI. This observation aligns with a study conducted by Han et al. (2013), which demonstrated that inulin with an average DP of 24 significantly increased cecal total lipid contents when compared to a cellulose-HFD in rats (Han et al., 2013).

Given that inulin is primarily fermented by intestinal microbiota rather than being absorbed by the host animal, we conducted *in vitro* experiments to examine the effects of inulin on certain bacteria. In our study, we found that inulin had the capacity to inhibit the growth of the pathogenic bacteria *E. coli*. Notably, this inhibitory ability increased with DP, which can be attributed to *E. coli*’s inability to utilize either long or short inulin as a carbon source due to the absence of crucial inulin degradation enzyme β-fructofuranosidases (Cockburn and Koropatkin, 2016). Additionally, HDI has been reported to form a stable gel in water (Bchir et al., 2019), which could potentially hinder nutrients uptake by *E. coli*.

Obesity is a significant risk factor in the development of T2D (Nagy and Einwallner, 2018). In our study, a high-fat diet indeed induced higher fasting blood glucose levels and impaired glucose tolerance, and these effects could not be reversed by the addition of inulin. The impact of inulin on insulin resistance is a topic of debate, with varying results reported in both animal experiments and human clinical trials (Han et al., 2013; Shao et al., 2020). This controversy is especially apparent in obese individuals, as insulin resistance may not be improved by inulin supplementation (Rao et al., 2019). It is noteworthy that obese mice exhibit a distinct composition of intestinal microbiota compared to lean mice, potentially accounting for the different effects on insulin resistance in obese and lean subjects. In our study, inulin was added 4 weeks after starting the high-fat diet, which differs from some other studies where inulin was supplemented from the beginning of the diet (Li et al., 2021), thus better reflecting its effects in obese individuals. Our findings suggest that glucose metabolism in the current study was not significantly influenced by inulin treatment, indicating that the observed changes in body weight due to inulin were not likely caused by alterations in glucose absorption.

To better understand why inulin reduced the body weight of high-fat diet-induced obese mice, we investigated the weight of white adipose tissue and brown adipose tissue. While there were no statistically significant differences among mice in the HFD + LDI, HFD + HDI, and HFD treatment groups, both LDI and HDI administration resulted in some reduction in white adipose tissue. UCP1 is responsible for thermogenesis in brown adipose tissue, which increases energy expenditure (Iwen et al., 2008). Notably, both LDI and HDI inulin increased the gene expression of *UCP1* in brown adipocytes by 200%, suggesting that LDI and HDI inulin could enhance energy expenditure in high-fat diet-induced obese mice, contributing to the observed reduction in body weight.

Furthermore, in our study, both LDI and HDI had a significant impact on the liver of obese mice when compared to white adipose tissues. These inulin treatments resulted in a reduction in lipid content in the liver, consistent with the observed decrease in liver weight, with HDI showing more pronounced effects. Triglycerides in the liver can originate from 3 main sources: uptake of fatty acids from the plasma (released by lipolysis), *de novo* synthesis of fatty acids, and dietary fat delivered by chylomicron remnants (Jensen-Urstad and Semenkovich, 2012). Since high-fat diet-induced insulin resistance was evident in our study, as indicated by high fasting blood glucose levels and reduced glucose tolerance, it is unlikely that the reduction in liver triglycerides was due to increased uptake of fatty acids from the plasma. As mentioned previously, inulin administration led to increased fecal fat excretion, which may have contributed to the reduction in dietary fatty acid uptake by the liver. Hepatic lipid metabolism is regulated by a dynamic transcriptional network, with nuclear receptors, particularly PPARs, playing a pivotal role. Srebp-1, highly expressed in the liver, regulates genes responsible for lipogenesis in response to insulin stimulation (Zhu et al., 2021).

Our results showed that HFD mice had the highest expression of the *Srebp1* gene, followed by inulin-administered mice and the LFD group. This pattern of gene expression correlates with the triglyceride contents in the treatment groups. Since *Srebp1* is regulated by insulin, the observed changes in its expression suggest that both LDI and HDI could improve insulin resistance caused by a high-fat diet. The reduced expression of *Srebp1* by LDI and HDI subsequently inhibited hepatic lipogenesis, leading to decreased hepatic triglyceride levels. The PPARα signaling pathway plays a crucial role in various metabolic processes, including fatty acid oxidation and ketone body synthesis. On the other hand, PPARγ is a key regulator of adipocyte differentiation, promoting adipogenesis and weight gain. Increased expression of *PPARγ* has been observed in patients with fatty liver disease (Skat-Rørdam et al., 2019). In our study, consistent with previous research, mice in the LFD group exhibited the lowest expression of the *PPARγ* gene, which was associated with the lowest hepatic triglyceride content. The addition of inulin inhibited the upregulation of *PPARγ* expression induced by the high-fat diet, resulting in reduced fat accumulation in the liver. Furthermore, overexpression of *PPARγ* has been reported to increase inflammation in the liver (Lee et al., 2018), which aligns with our findings of increased inflammation in the liver of HFD mice. Additionally, high-fat diet-induced elevation of *PPARγ* levels has been associated with increased expression of *Srebp-1*, a regulator of downstream lipogenic genes that enhances hepatic lipogenesis. Therefore, the high expression of PPARγ in the HFD group may have induced the activation of Srebp-1 and, in turn, increased hepatic lipogenesis. Besides regulating Srebp-1, PPARγ can directly target fat-specific protein, which is involved in both lipogenesis and fatty acid uptake in the liver. Thus, the high expression of PPARγ and Srebp-1 may have contributed to fat accumulation in the liver of HFD mice, while the reduced expression of these genes in LDI and HDI treatment groups implied a reduced capacity for lipid synthesis in the liver, consistent with the observed decrease in hepatic triglyceride content.

Obesity leads to defects in the mucus layer, characterized by increased penetrability and reduced mucus growth rate (Schroeder et al., 2020), which may result in the increased leakage of inflammatory substances into the body and contribute to increased inflammation in the liver, as observed in the HFD group of mice. Notably, the addition of HDI improved villus morphology in obese mice, suggesting a potential positive effect on certain aspects of intestinal function. Furthermore, gut microbiota plays a crucial role in maintaining intestinal health, and fiber consumption, such as inulin, is typically associated with increased diversity in intestinal microbiota in both animals and humans. Interestingly, in our study, the inclusion of inulin, especially HDI, significantly decreased the α diversity of cecal microbiota in high-fat diet-induced obese mice. This finding aligns with previous research that has also reported decreased α diversity in response to inulin supplementation, even at different doses and dietary contexts (Hutchinson et al., 2023; Le Bastard et al., 2020; Li et al., 2021). High-fat diets have been shown to reduce microbial richness, the richness did not fully recover with HDI or LDI administration in our study. Moreover, the diversity within individual samples significantly decreased with HDI inulin administration. It is important to note that interpretations of α diversity can vary depending on the calculation method used, and the significance or value of these metrics may not always be straightforward (Koutoukidis et al., 2022; Shade, 2017).

Examining the relative abundance of intestinal microbiota revealed an increase in the abundance of Verrucomicrobiota at the phylum level and *Akkermansia* at the genus level in the HDI inulin group. This led to increased dominance within the microbial community, potentially resulting in reduced evenness and, consequently, decreased diversity (as indicated by reduced Shannon and Simpson index) in the HFD + HDI group.

The increased abundance of *Akkermansia* in the cecum of obese mice due to the addition of HDI is of particular interest, as *Akkermansia muciniphila* is considered a potential health-promoting bacterium (Shin et al., 2014). Administrating *Akkermansia* to HFD-induced obese mice could strengthen the tight junction and mucus barrier (Li et al., 2024). Previous studies have shown that high-fat diets tend to decrease the abundance of *Akkermansia* in the microbiota of mice compared to low-fat diets, making its increase through dietary intervention a valuable finding (Wang et al., 2020). It is worth noting that the impact of inulin on *Akkermansia* abundance can be influenced by various factors, including diet composition and the interactions among different microbial species. For instance, some studies have reported that inulin supplementation, especially in the context of high-fat diets, can increase the abundance of *Akkermansia* (Pérez-Monter et al., 2022). The mechanisms behind these changes in *Akkermansia* abundance are complex and may involve factors such as the influence of HDI on the gut mucosal layer, potential cross-feeding interactions with other bacteria, or the HDI’s effects on the overall gut microbiota composition. Interestingly, another biomarker genus, *Erysipelatoclostridium*, was positively correlated with *Akkermansia* in the HFD + HDI group. *Erysipelatoclostridium* has been associated with anti-inflammatory properties in obese mice (Roager et al., 2019; van Trijp et al., 2020) and was found to increase during *in vitro* fermentation experiments with human small intestinal microbiota.

Overall, these findings suggest that dietary interventions with HDI may have a positive impact on gut microbiota composition, particularly with regard to the abundance of beneficial bacteria like *Akkermansia*, which could contribute to improved host health. Further research is needed to fully understand the complex interactions within the gut microbiota and how dietary factors like inulin can influence these interactions and promote health.

The correlations between specific gut microbiota and various metabolic and health parameters in response to inulin treatment provide valuable insights into the complex interplay between diet, gut microbiota, and host health. In the HFD group, the biomarker microbiota like *Colidextribacter*, Lachnospiraceae, Lachnospirales, and Dubosiella exhibited strong positive correlations with factors associated with obesity and fatty liver, such as body weight, triglycerides, *PPARγ*, and *Srebp1*. These correlations suggest that these microbiota may contribute to the development of obesity and its associated metabolic complications. The negative correlation with *UCP1* further highlights their potential role in reduced energy expenditure. Conversely, in the HFD + HDI group, the biomarker microbiota *Akkermansia* and *Erysipelatoclostridium* displayed positive correlations with *UCP1*, indicating a potential role in promoting energy expenditure. This is consistent with the observed reduction in body weight in this group. Gut microbiota plays vital roles in alleviating steatotic liver through reducing hepatic PPARγ and related lipogenesis genes (Murakami et al., 2016). The negative correlations with factors like body weight, *PPARγ*, and *Srebp1* in our experiment further suggest that these microbiota may play a role in reducing fat accumulation and improving liver health. The role of SCFA produced by gut microbiota, especially *Akkermansia*, is highlighted as a potential mechanism for improving liver health and reducing hepatic *PPARγ* expression. Additionally, aside from the potential metabolites that regulate hepatic *PPARs* expression and other lipid metabolic genes, *A. muciniphila* has been reported to mitigate inflammatory factors, thus reducing hepatic lipogenesis (Ghaderi et al., 2022). These findings suggest that the microbiota’s metabolites, influenced by dietary fiber such as inulin, can have significant effects on host health. The variations in outcomes observed in different studies using inulin with varying DP and different dietary conditions underscore the complexity of the gut microbiota-host interaction (Yamaguchi et al., 2021). The individual’s baseline health status, along with diet and specific inulin properties, can all contribute to different responses in gut microbiota composition and metabolic outcomes.

## Conclusion

Overall, our experiment highlights the potential benefits of HD inulin in managing body weight, ameliorating liver health issues, and modulating the composition and structure of cecal microbiota in obese mice (Figure 10). The observed effects of HDI in promoting the abundance of *Akkermansia* in the intestines of obese mice are noteworthy, as previous research has demonstrated the beneficial effects of certain *Akkermansia* species supplementation on both liver and intestinal health. Additionally, the limited effects of LDI on cecal *Akkermansia* may suggest that the differing impacts of different DP of inulin on obese individuals largely originate from their effects on the intestinal *Akkermansia*. If HDI administration yields similar effects to those of prebiotic supplementation, it could offer a more practical approach for alleviating fatty liver disease and managing body weight, given the ease of handling dietary fiber compared to live bacteria.

Furthermore, while our study demonstrated the enrichment of *Akkermansia*, it did not directly link this to improvements in intestinal and hepatic health. Further investigation is warranted to provide more robust evidence and to explore the mechanisms through which HD inulin could enrich *Akkermansia*. Additionally, more studies should be conducted to elucidate the specific metabolites responsible for these potential effects. Since inulin fiber is primarily utilized by intestinal bacteria, it is essential to identify the metabolites produced by these bacteria and their mechanisms of action. Additionally, conducting fecal bacteria translocation experiments in HFD-induced mice could provide further insights into the role of the microbiota in mediating the effects of inulin supplementation. These future investigations will contribute to a more comprehensive understanding of the mechanisms underlying the beneficial effects of HDI inulin on host health.

## Data Availability

The original contributions presented in the study are publicly available. This data can be found here: https://www.ncbi.nlm.nih.gov/, accession number PRJNA1151349.
