# Supplementary material for: The impact of high polymerization inulin on body weight reduction in high-fat diet-induced obese mice: correlation with cecal Akkermansia
Source: Front Microbiol. 2024 Aug 29;15:1428308. doi: 10.3389/fmicb.2024.1428308 (PMC11392436; doi:10.3389/fmicb.2024.1428308)
Supplement: Supplementary file 1 [file Data_Sheet_1.docx]

**Supplementary data**

Table S1 Ingredients of the high fat and low fat diet.

| Item | High fat diet | Low fat diet |
| --- | --- | --- |
| Casein L-cystine, g | 276 | 194 |
| Carbohydrate dextrin sucrose, g | 250 | 0 |
| Corn starch, g | 0 | 673 |
| Lard, g | 341 | 0 |
| Soybean oil, g | 0 | 40 |
| Fiber (Cellulose), g | 68 | 48 |
| Mineral and vitamin mixture, g | 65 | 45 |
| Antioxidant (TBHQ), g | 0.07 | 0.01 |
| Total, g | 1000 | 1000 |
| Energy，kcal/g | 5.1 | 3.6 |
| % kcal from Protein | 19% | 19% |
| % kcal from Carbohydrate | 21% | 71% |
| % kcal from Fat | 60% | 10% |

Table S2 Primer sequences used for qRT-PCR

| Name |  | Sequences 5’-3’^1^ | Source |
| --- | --- | --- | --- |
| UCP-1 | F^2^ | GGCCCTTGTAAACAACAAAATAC | Li, *et al.*, 2022 ^[3]^ |
|  | R | GGCAACAAGAGCTGACAGTAAAT |  |
| PPARα | F | CGTCACGGAGCTCACAGAAT | Clifford, et al., 2021 ^[4]^ |
|  | R | ACTCGCGTGTGATAAAGCCA |  |
| Mafg | F | GACCCCCAATAAAGGAAACAA |  |
|  | R | TCAACTCTCGCACCGACA |  |
| Scd1 | F | CCGGAGACCCCTTAGATCGA |  |
|  | R | TAGCCTGTAAAAGATTTCTGCAAACC |  |
| Lpin1 | F | CCTTCTATGCTGCTTTTGGGAACC |  |
|  | R | GTGATCGACCACTTCGCAGAGC |  |
| Dgat2 | F | GGCGCTACTTCCGAGACTAC |  |
|  | R | TGGTCAGCAGGTTGTGTGTC |  |
| Srebp1c | F | GGAGCCATGGATTGCACATT |  |
|  | R | GGCCCGGGAAGTCACTGT |  |
| Pgc1α | F | AAACTTGCTAGCGGTTCTCAC |  |
|  | R | GGCAATCCGTCTTCATCCAC |  |
| PPARγ | F | GCCCTTTGGTGACTTTATGG |  |
|  | R | CAGCAGGTTGTCTTGGATGT |  |
| β-actin | F | GTTGGTTGGAGCAAACATC |  |
|  | R | CTTATTTCATGGATACTTGGAATG |  |

^1^ Primer sequences are displayed in the 5’ to 3’ direction.

^2^ F, forward primer; R, reverse primer.

Table S3 The dimension of particle of inulin with different polymerized degree

| No. | Diameter of inulin powder, μm | |
| --- | --- | --- |
|  | Low polymerized degree | High polymerized degree |
| 1 | 77.733 | 24.765 |
| 2 | 76.126 | 23.112 |
| 3 | 68.653 | 24.753 |
| 4 | 74.019 | 20.138 |
| 5 | 96.099 | 19.567 |
| 6 | 79.508 | 29.68 |
| 7 | 86.551 | 10.415 |
| 8 | 159.816 | 19.453 |
| 9 | 106.815 | 11.609 |
| 10 | 104.564 | 21.958 |
| 11 | 104.666 | 20.198 |
| 12 | 67.165 | 10.415 |
| 13 | 132.868 | 21.307 |
| 14 | 66.848 | 12.798 |
| 15 | 74.823 | 19.377 |
| 16 | 66.509 | 14.608 |
| 17 | 74.019 | 8.313 |
| 18 | 59.057 | 9.969 |
| 19 | 42.517 | 12.964 |
| 20 | 50.78 | 7.579 |
| 21 | 52.807 | 19.839 |
| 22 | 76.907 | 11.035 |
| 23 | 41.053 | 14.445 |
| Average | 79.996 | 16.882 |

Table S4 The detailed wavenumber of FTIR spectra of inulin with different degree of polymerization

| Low degree of polymerization inulin | | |  | High degree of polymerization inulin | | |
| --- | --- | --- | --- | --- | --- | --- |
| Peak position, cm-1 | Intensity | interpretation |  | Peak position, cm-1 | Intensity | interpretation |
| 599.22 | 0.138 | OH out-of-pla^[1]^ne bend |  | 598.40 | 0.176 | OH out-of-plane bend |
| 781.48 | 0.0634 | -(CH_2_)- rocking |  | 821.70 | 0.114 | 2-ketose |
| 824.84 | 0.096 | 2-ketose^[2]^ |  | 934.06 | 0.344 | OH bending |
| 868.18 | 0.0779 | OH bending |  | 1027.59 | 0.787 | C-O-C stretching |
| 931.45 | 0.196 | OH bending |  | 1133.10 | 0.562 | C-O stretching |
| 1028.74 | 0.572 | C-O-C stretching |  | 1459.03 | 0.321 | C-H bending |
| 1109.68 | 0.402 | C-O stretching |  | 1648.40 | 0.136 | OH bending |
| 1269.26 | 0.204 | C-O stretching |  | 2934.59 | 0.362 | C-H stretching |
| 1340.12 | 0.211 | C-H bending |  | 3385.30 | 0.671 | O-H stretching |
| 1457.07 | 0.251 | C-H bending |  |  |  |  |
| 1646.91 | 0.106 | OH bending |  |  |  |  |
| 2934.90 | 0.285 | C-H stretching |  |  |  |  |
| 3386.55 | 0.58 | O-H stretching |  |  |  |  |

Table S5 The significantly differences of cecal microbiota at different taxonomic levels in mice of different treatment groups

| Microbiota | | Relative Abundance | | | | | |
| --- | --- | --- | --- | --- | --- | --- | --- |
|  |  | LFD | HFD+HDI | HFD+LDI | HFD | SEM | *P* value |
| **Phylum** | |  |  |  |  |  |  |
|  | Verrucomicrobiota | 0.0055 b | 0.2406 a | 0.0781 b | 0.0001 b | 0.00007 | 0.009 |
|  | Fusobacteriota | 0.00069 a | 0.00033 ab | 0.000003 b | 0.000074 b | 0.02265 | 0.005 |
|  | Firmicutes/Bacteroidota | 1.0939 | 2.2978 | 1.6823 | 2.8711 | 0.28637 | 0.136 |
| **Family** | |  |  |  |  |  |  |
|  | Akkermansiaceae | 0.005464 b | 0.240579 a | 0.078111 b | 0.000128 b | 0.0227 | 0.021 |
|  | Fusobacteriaceae | 0.000688 a | 0.000326 ab | 0.000003 b | 0.000074 b | 0.0001 | 0.034 |
| **Genus** | |  |  |  |  |  |  |
|  | Akkermansia | 0.005464 b | 0.240579 a | 0.078111 b | 0.000128 b | 0.022651 | 0.013 |
|  | Bilophila | 0.000115 b | 0.000269 b | 0.002074 a | 0.000068 b | 0.000188 | 0.045 |
|  | Candidatus_Soleaferrea | 0.000053 b | 0 b | 0 b | 0.000348 a | 0.00002 | 0.006 |
|  | Cetobacterium | 0.000688 a | 0.000326 ab | 0.000003 b | 0.000074 b | 0.00007 | 0.033 |
|  | Colidextribacter | 0.022275 b | 0.016398 b | 0.018153 b | 0.083236 a | 0.00821 | 0.033 |
|  | GCA_900066575 | 0.001825 b | 0.009164 ab | 0.001404 b | 0.024647 a | 0.002837 | 0.0499 |
|  | Harryflintia | 0.000513 a | 0.000027 b | 0 b | 0.000509 a | 0.00008 | 0.045 |
|  | Incertae_Sedis | 0.000409 ab | 0.001214 ab | 0.002867 a | 0.000174 b | 0.000334 | 0.043 |
|  | Negativibacillus | 0 b | 0 b | 0 b | 0.000373 ab | 0.00002 | 0.014 |
|  | unclassified_Christensenellaceae | 0.000223 b | 0.000053 b | 0.000025 b | 0.001333 ab | 0.00015 | 0.049 |
|  | unclassified_Clostridia | 0.000267 b | 0.000631 ab | 0.001613 a | 0.000197 b | 0.000192 | 0.045 |
|  | unclassified_Erysipelotrichaceae | 0.003598 a | 0 b | 0.000524 b | 0.002224 ab | 0.000512 | 0.044 |
|  | unclassified_Lachnospiraceae | 0.080019 a | 0.091354 a | 0.219439 b | 0.198044 b | 0.023523 | 0.039 |
| **Species** | |  |  |  |  |  |  |
|  | Ruminococcaceae_bacterium_GD6 | 0.000053 b | 0 b | 0 b | 0.000348 a | 0.00002 | 0.008 |
|  | unclassified_Akkermansia | 0.005464 b | 0.240579 a | 0.078111 b | 0.000128 b | 0.022651 | 0.017 |
|  | unclassified_Cetobacterium | 0.000688 a | 0.000326 ab | 0.000003 b | 0.000074 b | 0.00007 | 0.041 |
|  | unclassified_Colidextribacter | 0.020313 b | 0.016105 b | 0.018083 b | 0.078622 a | 0.007593 | 0.034 |
|  | unclassified_Lachnospiraceae_FCS020_group | 0.000063 a | 0.000943 b | 0.000628 ab | 0.000296 a | 0.000126 | 0.0498 |
|  | unclassified_Negativibacillus | 0 b | 0 b | 0 b | 0.000373 a | 0.00002 | 0.017 |

Within each row, means without a common letter differ significantly at *P* < 0.05.


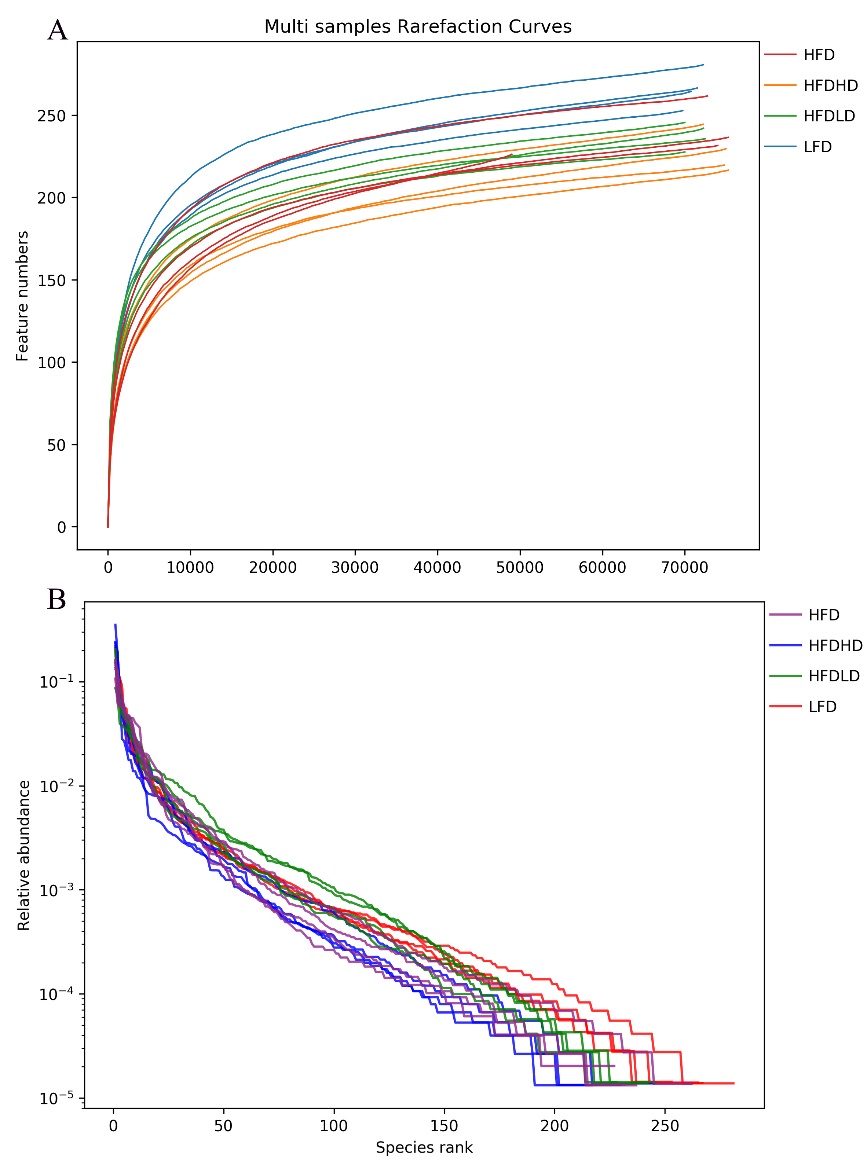


Figure S1 Rarefaction curves (A) and rank abundance plots (B) of cecal microbiota for all samples across different treatment groups. Abbreviations: HFD, high-fat diet treatment group mice; HFDHD, high-fat diet and high-degree polymerization inulin treatment group; HFDLD, high-fat diet and low-degree polymerization inulin treatment group; LFD, low-fat diet treatment group.


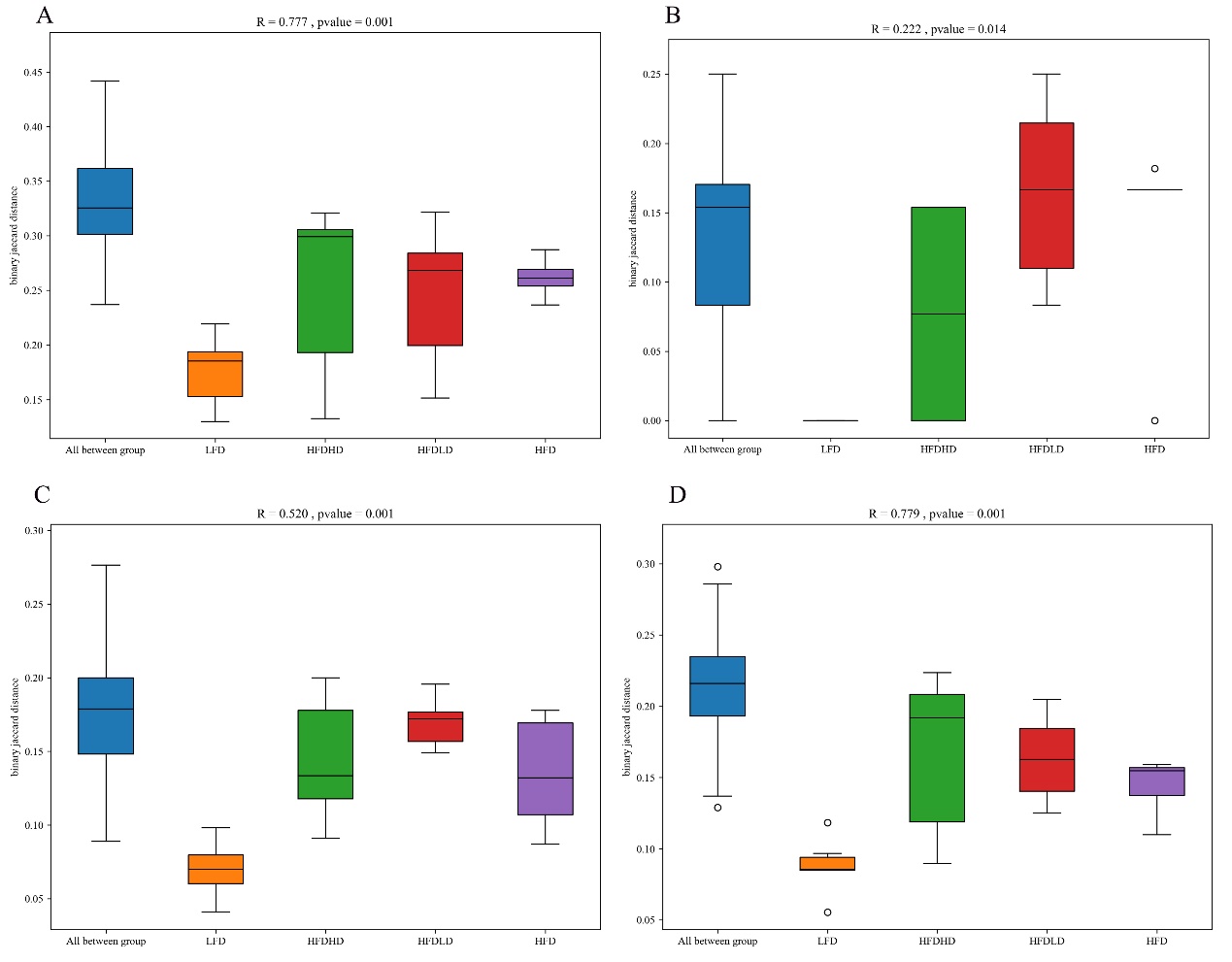


Figure S2 Analysis of similarities (ANOSIM) at the operational taxonomic unit (OTU) level (A), phylum level (B), family level (C), and genus level (D). An R value greater than 0 indicates no significant variations within treatment groups, while a *P* value less than 0.05 suggests significant differences among treatment groups regarding β diversity. HFD, high-fat diet treatment group mice; HFDHD, high-fat diet and high-degree polymerization inulin treatment group; HFDLD, high-fat diet and low-degree polymerization inulin treatment group; LFD, low-fat diet treatment group.

REFERENCE

[1] COATES J (2000). Interpretation of infrared spectra, a practical approach.

[2] AKRAM W, GARUD N. Optimization of inulin production process parameters using response surface methodology[J]. Future Journal of Pharmaceutical Sciences,2020, 6(1).

[3] LI Y, WANG D, PING X, et al. Local hyperthermia therapy induces browning of white fat and treats obesity[J]. Cell,2022.

[4] CLIFFORD B L, SEDGEMAN L R, WILLIAMS K J, et al. FXR activation protects against NAFLD via bile-acid-dependent reductions in lipid absorption[J]. Cell Metabolism,2021.
